# Simulation tools for particle-based reaction-diffusion dynamics in continuous space

**DOI:** 10.1186/s13628-014-0011-5

**Published:** 2014-10-24

**Authors:** Johannes Schöneberg, Alexander Ullrich, Frank Noé

**Affiliations:** 1 Department of Mathematics, Computer Science and Bioinformatics, Free University Berlin, Arnimallee 6 14195, Berlin, Germany

**Keywords:** Reaction-diffusion, Brownian dynamics, Particle simulation, Confinement, Excluded volume, Crowding

## Abstract

Particle-based reaction-diffusion algorithms facilitate the modeling of the diffusional motion of individual molecules and the reactions between them in cellular environments. A physically realistic model, depending on the system at hand and the questions asked, would require different levels of modeling detail such as particle diffusion, geometrical confinement, particle volume exclusion or particle-particle interaction potentials. Higher levels of detail usually correspond to increased number of parameters and higher computational cost. Certain systems however, require these investments to be modeled adequately. Here we present a review on the current field of particle-based reaction-diffusion software packages operating on continuous space. Four nested levels of modeling detail are identified that capture incrementing amount of detail. Their applicability to different biological questions is discussed, arching from straight diffusion simulations to sophisticated and expensive models that bridge towards coarse grained molecular dynamics.

## Introduction

Biological function relies on molecular reactions. In order for them to occur, the educts have to be in physical proximity, a situation often termed encounter complex. Depending on the reaction rate, this encounter complex then either, with a certain probability, reacts to products or dissociates again by diffusion. The overall probability of a reaction to occur therefore depends on two parameters: 1) the frequency by which the educts form encounter complexes and 2) the transition rate of this encounter complex to the actual products. While 2) depends on the detailed chemical and biophysical interactions between educt molecules, 1) depends on the properties of the molecular transport process (here diffusion) and the environment of this transport (crowding and obstructing cellular geometry). Reactions for which the chemical processes are fast compared to the transport are known as diffusion limited reactions. But essentially all molecular reactions depend on the circumstances of educt encounter to some degree [1]–[3].

A detailed computational approach to study the influence of environment and diffusion on reactions is that of conducting particle-based reaction-diffusion (PBRD) simulations. In such an approach, the molecular species of interest are modeled as individual particles that are transported via stochastic dynamics (usually Brownian motion) in continuous space and undergo reactions when certain criteria are met. Current computational tools model the particles and their interactions (with each other and the environment) on different levels of detail. This leads to an applicability to different areas of biological questions. In this paper we review software tools for conducting PBRD simulations with respect to the level of modeling detail. It should serve as a guide to choose a simulation tool that is appropriate for the modeling question at hand.

## Four levels of modeling detail

In this review we will specifically concentrate on approaches, that propagate particle positions explicitly in continuous physical space (as opposed to lattice based approaches) and allow reactions between them. These approaches include the following ten tools: CDS [4], Cell++ [5], ChemCell [6], eGFRD [1] (derived from GFRD [7],[8] and E-Cell [9]), Klann et al. [10],[11], MCell [12],[13], ReaDDy [14], Rigdway et al. [15], Smoldyn [16],[17] and SRSim [18].

The tools discussed here allow the modeling of reaction-diffusion systems on different levels of detail. The more a system shifts from a homogeneous single-compartment model, that is sparsely populated and where particle-particle forces can be neglected, towards an inhomogeneous multi-compartment model, that is crowded and in which particle-particle forces influence the dynamics, the more physical concepts have to be captured by the simulation tool and the more computationally expensive become the calculations. Such differences in calculation time for different levels of modeling detail can reach several orders of magnitude.

We will introduce the following four terms describing the level of modeling detail for a reaction-diffusion system. The higher levels thereby include all lower levels.

### (1) Free diffusion

Free diffusion is considered the basic level of detail. Particles (i.e. points on this level) diffuse in continuous space in a single compartment (i.e. the simulation box) according to Brownian motion. If particles come closer than a certain interaction distance, they might undergo a reaction. All ten simulation tools provide this level of modeling detail.

### (2) Confined diffusion

Additionally to particles undergoing free diffusion, this level of detail provides concepts of cellular geometry: Particles might not only diffuse in free space (3D) but also on 2D planes (i.e. membranes) or along channels (1D) and might be confined to compartments. On this level of detail, the diffusion of particles on or within the geometry structures should be provided. Also, reactions between particles confined to different geometries (e.g. uptake of a cytosolic ligand from a membrane bound receptor), and between particles and geometries themselves (e.g. adsorption of a cytosolic particle to a membrane) should be possible. Simulators on this level of detail include Cell++ [5], ChemCell [6], MCell [12],[13] and Smoldyn [16],[17]. Confinement is realized differently in Cell++ than in the other tools. Cell++ operates on cubic lattice rectangles that might be accessible or forbidden for particles. In this way a geometry is constructed from these building blocks. Actual 2D diffusion is not supported. The other tools provide three and two dimensional geometry building blocks of different shapes that particles are repelled from or confined to, including e.g. membranes.

### (3) Excluded volume

So far, the concept of a particle has been a point without volume extension. Real molecules however are volumetric objects that are not allowed to overlap and therefore generate ‘excluded volume’.

Considering diffusion, excluded volume leads to crowding that might influence the system’s apparent diffusion mobility considerably. Depending on particle density and mobility [19],[20], if there are mixed fractions of particles being mobile and immobile [21] and the size of the particles [22], either normal diffusion but with a decreased diffusion constant or anomalous diffusion [23]–[25] with a time dependent diffusion constant is observed. Experimentally observed apparent diffusion constants have to be adjusted to these effects which become accessible by modeling excluded volume. Considering reactions, excluded volume leads to the fact, that reaction rates have to be computed differently. Point particles are usually modeled as reactive spherical volumes. With particle excluded volume, this is no longer possible and the spherical reaction volume is replaced by a spherical reaction shell with a diffusion excluded interior. Excluded volume effects can have profound impact on a reaction-diffusion system as shown in [3],[15],[26],[27]. Excluded volume is provided in CDS [4], eGFRD [1], Klann et al. [10], Rigdway et al. [15], SRSim [18] and ReaDDy [14].

### (4) Particle-particle potentials

Many software tools implement excluded volume based on move rejection. If a particle move is attempted, the simulation engine checks for particle overlaps and rejects the move if an overlap would have occurred. This concept corresponds to particles modeled as hard-core repulsive spheres. Yet interactions of biomolecules are governed by an energy landscape that can be modeled with greater amount of detail. Examples are soft-core repulsive spheres, Van der Waals spheres or even a microscopic potential energy landscapes including all or many Van der Waals and electrostatic interactions between different molecules. An example of a diffusion simulation tool that is not focused on reactions but includes a representation of the molecular potential energy landscape is SDA [28],[29].

The modeling of particle-particle potentials becomes more important when attractive forces play a role in the reaction-diffusion system. It has been shown that clustered molecules lead to different reaction-diffusion dynamics than individual molecules [2],[30]–[32]. If attractive particle-particle potentials are part of a reaction-diffusion simulation, spatio-temporal processes such as molecular cluster formation and dissociation can be added to the picture.

Particle-particle potentials do not only play an important role for excluded volume and clustering effects. Harmonic potentials between particles can be used to model large complicated geometries built from individual particles. Connecting the introduction of such bonds with reactions allows the study of oligomerization and scaffold formation.

Particle-particle interaction potentials allow level 4 simulation tools to provide a bridge to Molecular Dynamics (MD) simulations. Parameters (e.g. the potential of mean force between two molecules) inferred from MD simulations can be used on the reaction-diffusion level to investigate the large space and long time scale behavior of high copy numbers. To our knowledge, SRSim [18] and ReaDDy [14] are the only tools that allow for particle-particle potentials in a reaction diffusion-simulation.

**Figure 1:**
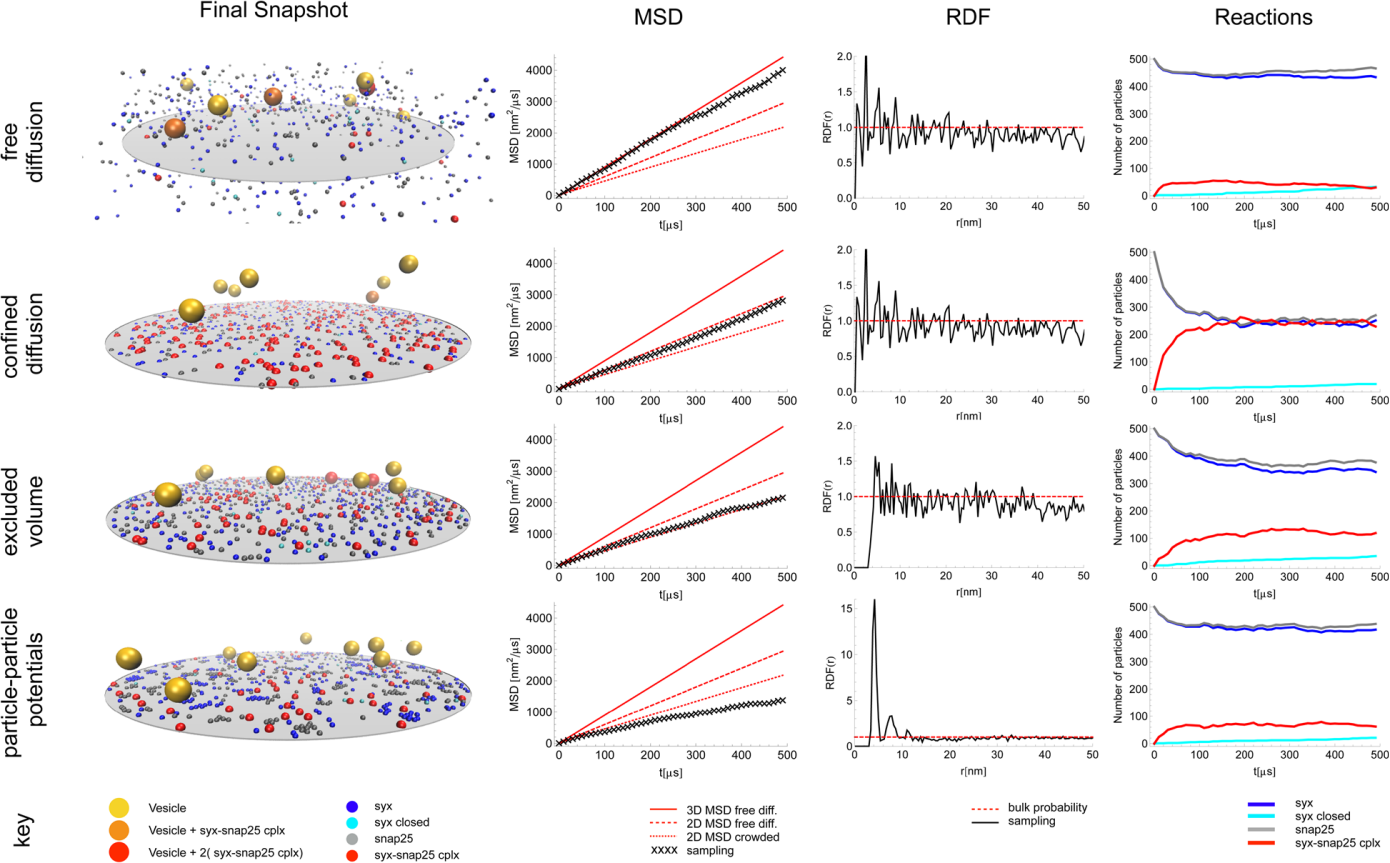
**Possibilities on the four levels of modeling detail.** The figure illustrates the four nested levels of modeling detail (rows) of particle-based reaction-diffusion simulation and the effects than can be captured on each level (columns). The *free diffusion level* (level 1) contains basic 3D diffusion of point particles and reactions between them in a simulation box. The *confined diffusion level* (level 2) adds the definition of cellular geometry and diffusion in compartments. The *excluded volume level* (level 3) replaces point particles with volumetric entities. The *particle-particle potential level* (level 4) allows the definition of potentials between particles. The levels are shown using an illustration model of synaptic vesicle release (the tutorial example of ReaDDy [14]) that demands all four levels of modeling detail: Syntaxin (syx, blue, cyan), snap25 (grey) and syx-snap25 (red) are bound to a 2D disk membrane (gray disk) while synaptic vesicles (large, yellow, red, orange) diffuse in 3D cytoplasm. Self-clustering of syx and snap25 are modeled by an attractive particle-particle potential. The same simulation parameters are used on each level. The columns show: A snapshot of the simulated trajectory, the mean squared displacement (MSD) evolution of syx, the radial distribution function (RDF) of syx, the particle number evolution in time due to reactions and the software packages available on each level. On level 1, pure 3D diffusion prevents the modeling of membrane bound proteins. Level 2 allows 2D confinement. Level 3 prevents particle-particle overlaps, visible in the RDF. Level 4 allows to model syx-syx attraction (the resulting clustering appears as solvation shells in the RDF). Note, the non-trivial influence of confinement, excluded volume and clustering on the apparent diffusion constant (i.e. slope of MSD) and the reactions, underlining the importance to choose the correct level of modeling for the biological system at hand.

The four levels of modeling detail and the effects that can be captured on the different levels are summarized in Figure 1.

## Comparison of simulation tools

Based on the four levels of modeling detail, introduced in the previous section, the ten particle-based reaction diffusion packages are compared. The results are summarized in Table 1.

**Table 1:** Particle-based reaction-diffusion simulation tools in continuous space

**Tool**	**Cell++**	**ChemCell**	**MCell**	**Smoldyn**	**CDS**	**eGFRD**	**Klann**	**Rigdway**	**SRSim**	**ReaDDy**
Detail Level	1/2	2	2	2	3	3	3	3	4	4
Dynamics	MC BD	MC BD	MC BD	MC BD	MC BD	GFRD	MC BD	MC BD	odL BD	odL BD
Time treatment	fixed steps	fixed steps	fixed steps	fixed steps	event-based	event-based	fixed steps	fixed steps	fixed steps	fixed steps
BD dimensions	3	2, 3	2, 3	1, 2, 3	1, 2, 3	2, 3	1, 2, 3	3	3	1, 2, 3
Particles	points	points	points	points	arb. volumetric	points	spheres, cylinders	spheres	sets ofspheres	sets ofspheres
Interaction p-p	-	-	-	-	-	-	-	-	potentials	potentials
Excluded volume	-	-	-	-	overlap rej.	overlap rej.	overlap rej.	overlap rej.	p-p repulsion	p-p repulsion
Geometry	constraints	constraints	constraints	constraints	constraints	constraints	constraints	constraints	constraints	potentials
Geo building blocks	cubes	cubes, planes	triangles	arb.	triangles	cubes, planes	arb.	cubes	cubes	arb.
Reaction dim.	3	3	2, 3	1, 2, 3	2, 3	2, 3	3	3	3	1, 2, 3
Reactions p-p	cubic subvol.	sph. subvol.	ray tracing subvol.	sph. subvol.	p-p collision	p-p collision	p-p collision	p-p collision	sph. subvol.	p-p collision
Reactions p-geo	-	-	ray tracing subvol.	sph. subvol.	p-geocollision	-	p-geocollision	p-geocollision	-	p-geocollision
First published	2006	2003	1996	2004	2010	2010	2011	2006	2010	2013
Availability	GNU GPLv2	GNU GPL	GNU GPLv2	GNU LGPL	Open Source	GNU GPL	-	-	GNU GPL	BSD 3 cl.
Reference	[5]	[6]	[12],[13]	[16],[17]	[4]	[1]	[10]	[15]	[18]	[14]

The level of modeling detail offered by the different tools is shown in the second row (1: free diffusion, 2: confined diffusion, 3: volume exclusion, 4: particle-particle potentials, see text), as well as their algorithmic details and availability. Web links: Cell++: www.compsysbio.org/lab/cellpp_details, ChemCell: chemcell.sandia.gov, M-Cell: www.mcell.org Smoldyn: www.smoldyn.org, CDS: nba.uth.tmc.edu/cds, eGFRD: www.gfrd.org, Klann:-, Rigdway: -, SRsim: www.biosys.uni-jena.de/,Members/Gerd+Gruenert/SRSim.html ReaDDy: www.readdy-project.org. *Abbreviations*: *MC BD* Monte Carlo Brownian dynamics, *GFRD* Greens Function Reaction Diffusion, *odL BD* overdamped Langevin Brownian dynamics.

Cell++, as described in [5], operates on point particles that can diffuse and react in continuous space in three dimensions (3D). Reactions are treated via a cubic subvolume method that is a derivative of the spatio-temporal master equation, sometimes also referred to spatial Gillespie [33]. Cell++ does not provide the possibility of higher level of modeling detail as it is defined in the previous section. It does however provide a unique ability that is not possible in any other packages listed here: Cell++ is able to represent some particle species as densities and some particle species as individual particles at the same time. This enables Cell++ to handle unique biological questions and to provide computational efficiency (since only a fraction of the particles are simulated individually). Cell++ is open source under the GNU GPLv2 license (www.compsysbio.org/lab/cellpp_details).

The three other tools available on detail level 2 are ChemCell [6], M-Cell [12],[13] and Smoldyn [16],[17]. All three algorithms rely on a stochastic, Monte Carlo based Brownian dynamics (MC BD) approach that uses fixed time steps and propagates point particles without volume exclusion. Geometries representing cellular structures can be introduced on the basis of constraints that are evaluated on every movement attempt of a particle. If a particle move-step would lead the particle to cross an impermeable barrier, a new particle position is generated. This new position is generated differently in the three algorithms. In ChemCell, the timestep fraction until collision is determined. The particle move is reverted and a new move is attempted, now only for the timestep fraction calculated before. If this move is successfull, a move is attempted for the remaining fraction of the total timestep. In both M-Cell and Smoldyn, the new particle position is generated based on ballistic ray reflection from the boundary. If particles are bound to a surface, their 2D diffusion trajectory is generated differently in the three approaches: ChemCell uses actual 2D diffusion. M-Cell is based on triangulated surfaces and allows only one particle per triangle. Surface diffusion is then simulated by a diffusion step that is taken towards the final position on the surface. If the tile at the target position is already occupied by a molecule, the move is rejected and retried. Smoldyn uses 3D diffusion to generate positions that are projected on the 2D surface to generate 2D diffusion. Reactions, as in level 1, are treated based on subvolumes. These subvolumes are spherical for both ChemCell and Smoldyn. M-Cell uses a cylindrical subvolume for reactions that is generated for each timestep between the start and the end position of the last particle move and the cross section of that particle. Potential reaction partners are molecules within this reaction cylinder and are discovered and tested for a reaction in temporal order (i.e. molecule closer to the starting point of the diffusion step are discovered before molecules close to the end of the trajectory). Particle-geometry reactions (e.g. membrane adsorption) are only possible in M-Cell and Smoldyn. On this level, all three tools are publicly available under an Open Source license (ChemCell: chemcell.sandia.gov, M-Cell: www.mcell.org, Smoldyn: www.smoldyn.org).

Excluded volume is supported in the following packages, as described in the respective publications: CDS [4], eGFRD [1], Klann et al. [10], Rigdway et al. [15], SRSim [18] and ReaDDy [14]. In the first four tools, excluded volume is implemented as an overlap rejection constraint, while it is implemented as a repulsive particle-particle interaction potential in SRSim and ReaDDy (see next section about particle-particle potentials). The overlap rejection constraints are enacted in different ways, due to the different underlying dynamics in the simulators. Klann et al. [10] and Rigdway et al. [15] implement Brownian dynamics with a fixed timestep. The overlap rejection operates on movement attempts which get rejected if the movement would have led to an overlap. Both CDS and eGFRD operate on event based adaptive timesteps. In this scheme the timestep is always chosen such, that particles can at most travel exactly next to each other to prevent overlaps (i.e. smaller timesteps if particles are close together). In all six algorithms, reactions are triggered with the actual collision of particles. Both CDS and eGFRD implement an extension of the Smoluchowski theorem for diffusion limited reactions including excluded volume [7],[8],[34],[35]. Klann et al. and Rigdway et al. derived their own algorithms for reaction probability calculation including excluded volume [10],[15].

Considering the runtime of these algorithms, the event based methods (CDS, eGFRD) are faster than other methods for cases of dilute medium. If the system is more crowded and the next collision between particles is always imminent, this advantage of event based timesteps is lower. On this level, only (http://nba.uth.tmc.edu/cds/index.htm) and eGFRD (www.gfrd.org) are publicly available.

Interaction potentials between particles are supported by SRSim [18] and ReaDDy [14]. Both tools model dynamics based on the overdamped Langevin equation in a potential. SRSim operates on the particle dynamics implementation of LAMMPS [36] while ReaDDy has its own core. Both tools feature particle-particle interaction potentials that are used in several ways: purely repulsive potentials capture excluded volume effects, weak attractive potentials between particles can model clustering effects, and attractive harmonic potentials between particles enable both tools to build larger oligomeric structures from individual spherical particles. In ReaDDy, the potential term in the Langevin equation is also used to model cellular geometry and confinement (e.g. 2D membrane potentials or spherical potentials that confine particles on a plane or a spherical surface). Reactions are modeled slightly different in SRSim and ReaDDy: In SRSim the number of simulated particles remains constant. A reaction, between two educt particles that form a product particle, is modeled by the introduction of a harmonic bond between the educts upon collision with a certain probability [18]. A special feature of SRSim is the possibility to take particle-particle orientation into account for reactions. The reaction system in ReaDDy is similar to the algorithms on the excluded volume level 3 and is based on the Erban and Chapman approach [35]. ReaDDy allows both concepts: Particles can get replaced due to a reaction but also a bond can be introduced between them. ReaDDy supports groups of particles that can react with each other. In this way a fine-graining of the modeled molecules is possible. This allows ReaDDy to model molecules as sets of spheres (i.e. groups) that are connected via harmonic potentials. In this set, only specific spheres might be reactive, allowing for the modeling of e.g. binding pockets and reactive patches. Both tools are publicly available (SRSim: www.biosys.uni-jena.de/Members/Gerd+Gruenert/SRSim.html, ReaDDy: www.readdy-project.org).

## The system selects the tool

To chose the right modeling tool for the system at hand is difficult. A main parameter that determines this decision is *computational cost*.

Level 4 reaction diffusion simulations allow capturing many physical concepts correctly (e.g. crowding, clustering, oligomerization) but are computationally expensive, placing them in the range of high performance computing. Compared to that, level 2 methods that lack interaction potentials and particle space exclusion, can easily be two to four orders of magnitude faster. As an example we compare the simulation performance of ReaDDy v1.1. and Smoldyn-2.29 on a cubic system of 100 nm edge length filled with 5000 particles at 30% packing density (roughly the density of cytoplasm [37]). The time per integration step on a single CPU was found to be ≈ 200 ms for ReaDDy and ≈ 2 ms for Smoldyn. If a typical integration timestep for ReaDDy is used (nanoseconds), systems of similar size can be simulated for up to milliseconds to seconds on level 4. Given the example above, one day of computation would result in ≈ 0.5 ms simulated time in ReaDDy and ≈ 50 ms for Smoldyn. However, these values strongly depend on the magnitude of diffusion constants and reaction rates, since they determine the timestep that has to be chosen. Advances in computing technology, such as parallel hard- and software (e.g. GPU computing) will reduce the wall-clock time of simulations. E.g. there is a GPU based parallel version of ReaDDy (Biedermann, Ullrich, Schöneberg and Noé: ReaDDyMM: fast particle-based reaction-diffusion simulations using graphical processing units, submitted) that is up to two orders of magnitude faster than the single core ReaDDy. Yet, the difference in modeling detail will remain to cause lower detail simulations to cost less than higher detail level simulations. Computational cost is also influenced by the density of the modeled system. If the system is dilute, event-based integration schemes (e.g. CDS or eGFRD) can be used to speed up the computation significantly. To give a rule of thumb: The number of particles usually determines the runtime of the portrayed simulators. Simulating more particles decreases the reachable runtime or the resolveable physical detail. One order of magnitude in the number of particles decreases the reachable runtime by the same order of magnitude, or alternatively the physical detail has to be decreased by one level. The first questions, when a system is to be modeled via PBRD should therefore be: How many particles are involved? What are the timescales of the processes at hand? How dense/sparse are the particles distributed? Can I afford to relax physical detail in order to gain computation speed?

The second aspect of choosing the right modeling tool is then its *toolbox*. Given a chosen level of physical detail, some tools provide the user with more help to incorporate complicated geometries or to setup extensive reaction networks easily. On level 2, M-Cell and Smoldyn offer the user help to setup, analyze and visualize simulations. With Libmoleculizer [38], Smoldyn offers a customized version of Moleculizer [39], a rule based reaction network generator and comes with a built-in tool for simulation trajectory visualization. The M-Cell team developed Cell-Blender, a plugin for the 3D computer graphics software Blender [40] in which new simulations can be setup, started and visualized, paired with the built-in 3D modeling tools of Blender. This allows to model even very complicated geometries easily in M-Cell. On level 3, CDS offers a sophisticated 3D simulation setup and visualization tool. eGFRD is embedded into the E-Cell framework, providing the user with the extensive simulation setup and evaluation toolbox of E-Cell [9]. On level 4, both ReaDDy and SRSim offer the user to build more fine grained particles, i.e. molecules can be modeled as a set of spheres held together by bonds. Due to the fact that geometry is modeled as potentials in ReaDDy, arbitrary geometries are possible.

In Summary, the choice of the computational tool is very system dependent. The system, with its number of particles, its timescales and its demand of physical detail level determines the computational cost. The combination of computational cost and the ease of use of the tool should then determine the choice.

## Application perspective

We close with proposing some perspective applications for PBRD simulations. Thereby, the introduction of more physical detail, from cellular geometry, over volume exclusion up to particle-particle interactions gives rise to interesting fields of applications.

### Compartmentalization and confinement

Compartmentalization of molecules, induced by cellular geometry can lead to the confinement of molecules in smaller subspaces. Such compartments can be very heterogeneous in terms of their shape (e.g. narrow tunnels [41]), molecule concentration and composition, thus leading to different reaction rates and behaviors as compared to bulk [42]. An example is the local depletion of a substrate in a sub-compartment although the substrate is highly abundant overall. Compartmentalization and localization also leads to an increased encounter rate between molecules. This becomes especially relevant if a reaction should be done in sequence, such as phosphorilation [1].

### Macromolecular crowding

Crowding is a complex phenomenon. Different particles are affected differently [43], and effects vary for different cell types [44]. Experiments usually have only a few types of crowders at hand to make quantitative statements. In silico experiments can be used to gain insight into the behavior and influence of crowding on both the diffusion and reactions at different levels of modeling detail. Crowding can be modeled through volume exclusion of static geometrical crowders (Level 2) and volume exclusion of particles, either through overlap rejection (Level 3) or particle-particle interactions (Level 4). The effects of volume exclusion on the diffusion of particles [45] and the contribution to association and reaction rates have been studied in several systems but are still debated [3],[15],[26],[27]. General insights on the influence of crowding on complex biological behavior can be provided by reaction-diffusion simulations.

An example is a study of the expression of a long term depression in synapses [46], where an unexpected non-linear influence of the crowding density was found that also agrees with previous experimental predictions. eGFRD and other tools have been used to investigate the influence of crowding on genetic networks [47]–[49] and active transport processes in cells [50]. In the same way, we can expect many reaction-diffusion systems to be affected by crowding. Simulating different crowding scenarios (different crowders and densities) could help to find general insights on the influence of crowding on complex biological behavior.

### Particle-particle potentials

Particle-particle potentials in reaction-diffusion simulations open new fields of investigation. Major examples are weak, transient interactions between molecules, clustering, molecular oligomerization and molecular shape.

**Figure 2:**
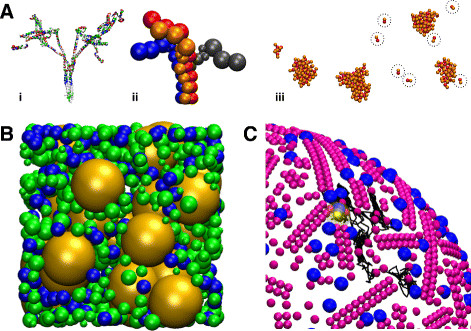
**Application perspective of particle-particle potentials in reaction-diffusion dynamics.** The incorporation of inter-particle potentials introduces several fields of applications. **(A)** Bond potentials allow the building of particle groups to form more realistic representations of proteins or other complexes of molecules (i: crystal structure of four Syntaxin-1a proteins (syx), ii: 11-particle representations of syx). iii: specific attraction potentials allow the study of self-organized syx cluster formation through weak homophilic interactions (dotted circles enclose individual syx). **(B)** The use of repulsive and weakly attractive potentials allows the study of reactions in highly crowded systems such as the cytosol. Depicted is a 100 nm cube of cytosol in which tRNAs (blue) and ribosomes (yellow) diffuse and react, together with all other molecules (green). **(C)** Groups of repulsive particles can be used to model the specific architecture [54],[55] of rhodopsin (purple) in the rod cell visual cascade. Photon activated rhodopsin (yellow) diffuses through the architecture (black line) and activates G proteins (blue). The depicted systems were modeled in ReaDDy [14] and visualized with VMD [56].

Transient, non-specific interactions between molecules are known to influence diffusion [51],[52]. A whole E-coli cytoplasm diffusion study, based on crystal structure shaped particles including their full potential energy landscape was done by Mc Guffee and Elcock [53] showing the formation of transient complexes with short lifetimes influencing the diffusion dynamics. The investigation of these effects on reactions between particles on a cytoplasm scale would be an interesting future application of level 4 reaction-diffusion simulations (compare Figure 2B for an illustration).

Clustering is a prominent biological process that involves weak interactions between particles [57]–[59] that might play a role in many systems with spatial organization [60]. Particularly the advent of super-resolution microscopy showed the phenomenon of self-organization through weak protein-protein interactions [61],[62]. Many receptors, such as chemotaxis sensing complexes, are known to form clusters. Their aggregation plays a crucial role in the chemotaxis signaling [63],[64] e.g. due to cooperative effects of the aggregated receptors [65] and the periodic spatial organization that arises through the cluster formation [66]. In general, many membrane microdomains, which are important units exerting specific biological function, might be formed solely via weak protein-protein interactions [61],[67] without the need of lipid rafts or cytoskeleton.

Syntaxin clustering is an interesting example of a membrane protein cluster formation in the synaptic vesicle exocytosis. A weak interaction between syntaxins leads to clusters that have been found to be about 50-60 nm wide and contain approximately 75 Syntaxin molecules [68]. About 15-30% of Syntaxins are freely diffusing while the rest belongs to clusters of different sizes. The large syntaxin clusters are almost immobile but highly dynamic in that they constantly exchange single Syntaxin molecules. The function of the clusters is not yet really understood. One likely role is that of a docking station for synaptic vesicles [69],[70]. PBRD simulations can be used to investigate the advantage of clustering over individually distributed molecules, e.g. as done with eGFRD [2]. In a recent study using ReaDDy, we show that the clustering of syntaxin via weak homophilic interactions can explain location specific functions of syntaxin as well as its localized clustering preference (Ullrich, Böhme, Schöneberg, Depner, Sigrist and Noé: Dynamical organization of Syntaxin-1A at the presynaptic active zone, submitted).

### Oligomerization and self assembly

Constructing oligomeric structures with a definite structure and stoichiometry is necessary in many biological processes. Metabolons, multiprotein complexes of enzymes of the same metabolic pathways, are an example. The spatial proximity of enzymes that act in sequence can enable a much higher throughput of the overall metabolic pathway [71]. G protein coupled receptors (GPCR), important cell signaling mediators, are known to occur in complexes. In the visual cascade, such organization is debated. Different oligomeric structures have been seen such as large clusters containing thousands of GPCRS [72] or racks of GPCR dimers that can occur in different lengths [54] (shown in Figure 2C). PBRD simulations can be used, not only to investigate the effects such structures would have on the diffusion and kinetics of signaling cascades ([73],[74]), but also how such structures would assemble [75],[76]. The possibility in level 4 reaction-diffusion simulations, to introduce a bond between particles in a polymerization reaction, will allow further study in this direction.

### Molecular shape

In most reaction-diffusion simulations, molecules are treated as spheres (if not as points). Ando and Skolnick showed, that the exact molecular shape can be neglected for diffusion simulations and be replaced by spheres [77]. However, for some molecular reaction-diffusion systems, a certain amount of fine-graining might be necessary, e.g. association of elongated, thread like proteins such as Syntaxin (see Figure 2A) or protein-ligand binding involving a distinct binding pocket. This pocket might be modeled as a reactive patch on an otherwise spherical particle, as it is implemented in SRSim. A comparable approach is used in ReaDDy where the protein would be modeled as a group of bonded spherical particles from which some are reactive. In this respect, a fine-grained reaction diffusion simulation that features particle-particle interaction potentials very much resembles coarse-grained atomistic simulation methods [78]. This bridge might allow for interesting future applications. While the costly atomistic calculations would only allow for the simulation of a few molecules over a nanosecond timescale [79], they could parametrize a coarser reaction-diffusion simulation that could investigate the interplay of hundreds of molecules for seconds.

## Competing interests

The authors declare that they have no competing interests.

## Authors’ contributions

JS, AU and FN did the research and together drafted and wrote the manuscript. All authors read and approved the final manuscript.
